# Limited Microbiota-Modulating Effects of Dietary Xylo-Oligosaccharide Supplementation in *Apis mellifera*

**DOI:** 10.3390/microorganisms14102208

**Published:** 2026-10-01

**Authors:** Ling Yin, Hongqing Fu, Ping Liu, Kang Wang

**Affiliations:** 1 Taizhou Key Laboratory of Food Biomanufacturing, School of Food Science and Health, Jiangsu Agri-Animal Husbandry Vocational College, Taizhou 225300, China; 2 College of Animal Science and Technology, Yangzhou University, Yangzhou 225000, China

**Keywords:** honey bees, xylo-oligosaccharides, prebiotic, gut microbiota

## Abstract

Prebiotics are widely used to modulate gut microbial communities, but their effects on the honey bee gut microbiota remain poorly understood. Xylo-oligosaccharides (XOS) are recognized as potential prebiotic substrates in humans and livestock, whereas their effects in honey bees have rarely been investigated in vivo. In this study, we evaluated the effects of dietary XOS supplementation on the gut microbiota of *Apis mellifera*. Total bacterial load and the absolute and relative abundances of five core bacterial groups (*Lactobacillus* Firm-5, *Bombilactobacillus*, *Bifidobacterium*, *Gilliamella*, and *Snodgrassella alvi*) were quantified using spike-in-based absolute 16S rRNA gene sequencing. Microbial diversity and community structure were further assessed using alpha-diversity indices and beta-diversity analyses. XOS supplementation did not significantly affect total bacterial abundance or the abundance of the five core bacterial groups (*p* > 0.05). No significant differences were detected in alpha diversity (*p* > 0.05), and the control and XOS groups exhibited largely overlapping community structures based on unweighted and weighted UniFrac analyses. However, PICRUSt2 analysis identified differences in several predicted microbial functional pathways between the two groups (*p* < 0.05). These results indicate that dietary XOS supplementation had limited detectable effects on the abundance, diversity, and structure of the established honey bee gut microbiota under the tested conditions, although changes in its predicted functional potential were observed. Our findings suggest that the efficacy of prebiotics in honey bees may depend on host-specific microbial ecology, and that prebiotic responses observed in mammalian systems may not be directly applicable to honey bees. These results highlight the importance of validating commercial prebiotic products using bee-specific in vivo evidence before their widespread application.

## 1. Introduction

Prebiotics are substrates that are selectively utilized by host-associated microorganisms and may thereby improve host health [1,2]. Most prebiotics are nondigestible carbohydrates that resist digestion by host enzymes but can be metabolized by specific members of the gut microbiota [3,4,5]. Through this process, prebiotics may influence microbial growth, community composition, and the production of fermentation metabolites [6,7,8]. Commonly studied prebiotics include inulin, fructo-oligosaccharides, galacto-oligosaccharides, and xylo-oligosaccharides, which have been widely investigated in humans and livestock [9,10]. Interest in microbiota-targeted dietary supplementation has also increased in mass-reared insects used for food, feed, and other commercial purposes. In these systems, manipulation of the gut microbiota through probiotic or prebiotic approaches has been proposed as a strategy to improve insect growth, nutrient utilization, health, and resistance to disease, although the available evidence remains much more limited than that in vertebrates [11].

The gut microbiota plays an important role in the nutrition, development, immunity, and overall health of honey bees [12,13,14,15]. Compared with the complex gut communities of many vertebrates, the adult honey bee gut microbiota is relatively simple and is dominated by a small number of host-associated bacterial lineages, including *Lactobacillus* Firm-5, *Bombilactobacillus*, *Gilliamella*, *Bifidobacterium*, and *Snodgrassella* [15,16]. These bacteria are consistently detected in healthy worker bees and form a stable microbial community within the ileum and rectum. Their restricted composition and close association with the host have made the honey bee gut microbiota a useful model for studying interactions among diet, gut bacteria, and host physiology [17,18].

Despite the well-established effects of prebiotics in humans and other animals, their application in honey bees has received comparatively limited attention. Current studies have primarily focused on their effects on host survival and pathogen resistance, particularly their potential to alleviate nosemosis [19,20,21,22,23]. However, one study reported that dietary microbial interventions involving prebiotics did not prevent nosemosis and, in some treatments, were associated with disrupted immune homeostasis and increased mortality [21]. Prebiotics have also been examined under in vitro conditions. In a fermentation system established using the gut microbiota of *Apis cerana*, inulin, fructo-oligosaccharides, xylo-oligosaccharides, and isomalto-oligosaccharides showed different effects on bacterial growth and acid production [23,24]. In addition, an in vivo study evaluated inulin supplementation in *Apis mellifera*, but no prebiotic effect was detected using the methods applied in that study [25]. Thus, direct evidence for the effects of individual prebiotics on the abundance, diversity, and community structure of the honey bee gut microbiota remains limited.

Xylo-oligosaccharides are short-chain oligosaccharides composed mainly of β-1,4-linked xylose units and are generally produced from xylan-rich plant materials [26,27]. Because XOS are resistant to digestion by host enzymes, they can reach the lower intestine and be utilized by specific members of the gut microbiota [27,28]. Previous studies in humans and livestock have shown that XOS supplementation can promote beneficial bacteria, particularly *Bifidobacterium*, and alter microbial fermentation and metabolite production [29,30,31]. However, the capacity to utilize XOS varies among bacterial species and strains, and its effects may depend on the composition of the resident microbiota and the experimental conditions [28,32]. Although XOS has been included in an in vitro study of the *Apis cerana* gut microbiota [24], its effects on the abundance and community structure of the honey bee gut microbiota in vivo remain unclear. In the present study, we evaluated the effects of dietary XOS supplementation on the gut microbiota of *Apis mellifera* by examining bacterial abundance, alpha diversity, and community structure. This study aimed to determine whether XOS could modulate the honey bee gut microbiota in vivo.

## 2. Materials and Methods

### 2.1. Honey Bees and XOS Preparation

Three healthy colonies of *Apis mellifera* were maintained at the experimental apiary of Yangzhou University, China. Xylo-oligosaccharides (catalog No. S11137; Shanghai Yuanye Biological Technology Co., Ltd., Shanghai, China) were dissolved in 50% (*w*/*v*) sucrose solution to a final concentration of 1% (*w*/*v*) [24]. The same 50% (*w*/*v*) sucrose solution without XOS was used for the control group (CK).

### 2.2. Experimental Design and XOS Supplementation

To obtain a synchronized cohort of newly emerged workers, the queen of each colony was confined to a single comb frame 18 days before the experiment. Brood frames were subsequently transferred to the laboratory and incubated at 35 °C and 90% relative humidity. Dark-eyed, late-stage pupae were aseptically removed from capped brood cells and individually placed in sterile 24-well tissue culture plates until emergence. Active workers emerging within 24 h were collected and placed in sterile rearing cages at 20 bees per cage, with three replicate cages established for each treatment.

The microbial inoculum was prepared from donor bees collected from the same three colonies. Briefly, three nurse bees with visibly full guts were selected from each colony. After aseptic dissection, the guts were pooled and homogenized with a sterile pestle. The homogenate was suspended in 800 μL of sterile phosphate-buffered saline, thoroughly mixed, and briefly centrifuged at low speed for 15 s to remove large debris. The resulting supernatant was used as the microbial inoculum. The microbial inoculum was incorporated into the pollen patty at a proportion of 100 μL microbial suspension per 1 g diet. The same inoculated pollen patties were provided to both the CK and XOS groups. Throughout the experiment, all bees were provided with pollen patties and the corresponding sucrose solution. The control group received 50% (*w*/*v*) sucrose solution, whereas the XOS group received 50% (*w*/*v*) sucrose solution containing 1% (*w*/*v*) XOS. Bees were maintained in a climate-controlled incubator at 30 °C and 60% relative humidity under continuous darkness. Dead bees were removed daily.

The 7-day feeding period was selected because the characteristic adult honey bee gut microbiota is generally established within approximately 6–7 days after emergence. After the 7-day feeding period, five bees were randomly selected from each cage, resulting in 15 bees per treatment. The bees were anesthetized with CO_2_ and surface-sterilized with 75% ethanol, followed by three rinses with sterile distilled water. Whole guts were aseptically dissected, immediately frozen in liquid nitrogen, and stored at −80 °C until DNA extraction. All 15 individual gut samples from each treatment were used for bacterial quantification and 16S rRNA gene sequencing.

### 2.3. Gut Microbiota Characterization

Genomic DNA was extracted using the FastDNA SPIN Kit (MP Biomedicals, Santa Ana, CA, USA). DNA integrity and concentration were assessed by agarose gel electrophoresis and Qubit fluorometry (Thermo Fisher Scientific, Waltham, MA, USA), respectively.

Gut bacterial communities were characterized using spike-in-based absolute 16S rRNA gene sequencing. Synthetic spike-in sequences retained the conserved bacterial primer-binding regions but contained artificial variable regions with approximately 40% GC content and no matches in public nucleotide databases. Nine spike-in standards covering a predefined concentration range were added to each DNA sample before amplification [33]. The bacterial 16S rRNA gene V3–V4 region and the spike-in standards were co-amplified using primers 341F and 805R [34]. Amplicons were purified with AMPure XP beads (Beckman Coulter, Brea, CA, USA), quantified using a Qubit fluorometer, pooled at equimolar concentrations, and sequenced on an Illumina NovaSeq 6000 platform (Illumina, San Diego, CA, USA) using 2 × 250 bp paired-end reads.

Sequence data were processed in QIIME 2 (version 2025.10) [35]. Primers and adapters were removed using Cutadapt (version 5.0), and reads were quality-filtered, denoised, merged, and checked for chimeras using DADA2 (version 1.36.0) [36]. Amplicon sequence variants were taxonomically assigned using a naive Bayes classifier trained on a honey bee gut microbiota reference database [37], with a confidence threshold of 80%. Relative abundances were calculated from the amplicon sequence variant table, whereas absolute abundances were estimated using the spike-in-derived total 16S rRNA gene copy numbers.

Alpha diversity was evaluated using Observed, Chao1, ACE, Shannon, Simpson, and Pielou’s evenness indices. Differences in community structure were assessed using unweighted and weighted UniFrac distances, visualized by non-metric multidimensional scaling, and tested by permutational multivariate analysis of variance. Microbial functional profiles were predicted from the 16S rRNA gene sequencing data using PICRUSt2 (version 2.3.0) (Phylogenetic Investigation of Communities by Reconstruction of Unobserved States 2). PICRUSt2 predicts the potential functional composition of bacterial and archaeal communities by linking taxonomic profiles derived from 16S rRNA gene sequences to reference genomes with known functional annotations, while accounting for variation in 16S rRNA gene copy number among taxa. The predicted functional profiles were subsequently used to compare potential metabolic pathways between the CK and XOS groups.

### 2.4. Statistical Analysis

Differences in bacterial abundance and alpha-diversity indices between the CK and XOS groups were analyzed using linear mixed-effects models (LMMs), with treatment included as a fixed effect and cage identity as a random effect to account for the non-independence of bees housed within the same cage, using the model lmer(Response ~ Treatment + (1|Cage)). Differences in overall microbial community structure were assessed by permutational multivariate analysis of variance (PERMANOVA) based on unweighted and weighted UniFrac distance matrices. Statistical analyses and data visualization were performed using SPSS 27.0 and R software (version 4.2.x). Differences were considered statistically significant at *p* < 0.05.

## 3. Results

### 3.1. XOS Supplementation Did Not Produce Detectable Changes in the Abundance of Major Gut Bacteria

**Figure 1:**
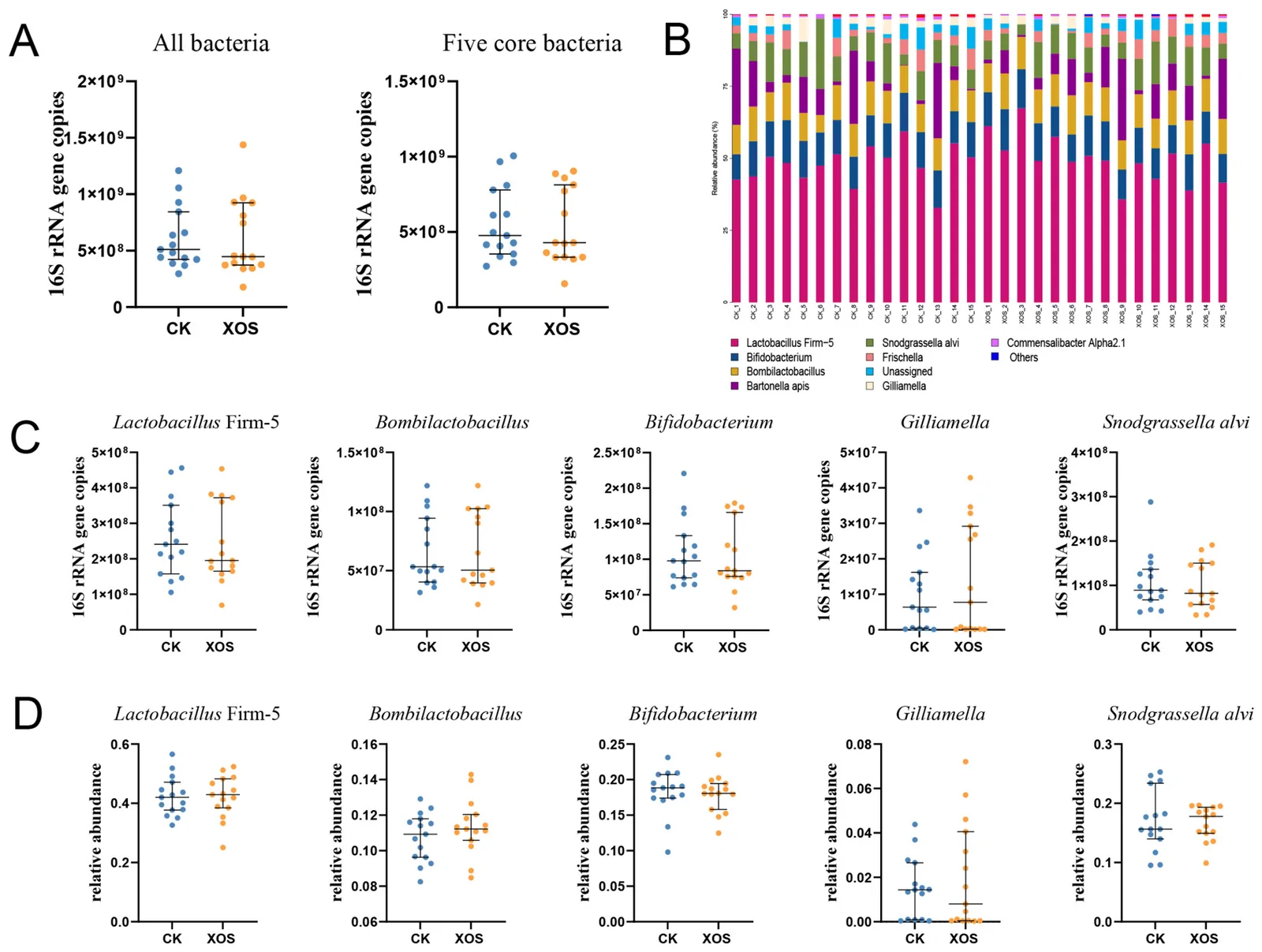
Effects of xylo-oligosaccharide supplementation on the abundance and composition of the honey bee gut microbiota. (**A**) Absolute abundance of total bacteria and the five core bacterial groups, expressed as 16S rRNA gene copies. (**B**) Relative abundance of the major bacterial taxa. (**C**) Absolute abundance and (**D**) relative abundance of *Lactobacillus* Firm-5, *Bombilactobacillus*, *Bifidobacterium*, *Gilliamella*, and *Snodgrassella alvi*. Each point represents one individual bee (*n* = 15). Horizontal lines indicate the median, and error bars indicate the interquartile range. CK, control group; XOS, xylo-oligosaccharide-supplemented group.

The total bacterial load and the combined abundance of the five core bacterial groups were similar between the CK and XOS groups (Figure 1A, *p* > 0.05). The gut microbiota in both groups was mainly composed of *Lactobacillus* Firm-5, *Bifidobacterium*, *Snodgrassella alvi*, *Bombilactobacillus*, and *Gilliamella*, and the overall community profiles showed no obvious separation between treatments (Figure 1B).

Analysis of individual bacterial groups further showed that XOS supplementation did not significantly affect the absolute abundance of *Lactobacillus* Firm-5, *Bombilactobacillus*, *Bifidobacterium*, *Gilliamella*, or *S. alvi* (Figure 1C, *p* > 0.05). Although *Gilliamella* varied considerably among individual bees, this variation was observed in both groups and did not form a consistent treatment-related pattern. Similar results were obtained for relative abundance, with no significant differences detected for any of the five core bacterial groups between the CK and XOS groups (Figure 1D, *p* > 0.05). Overall, no clear change was detected in either the total bacterial abundance or the composition of the major gut bacterial groups following XOS supplementation under these experimental conditions.

### 3.2. XOS Supplementation Did Not Produce a Detectable Change in the Alpha Diversity of the Honey Bee Gut Microbiota

**Figure 2:**
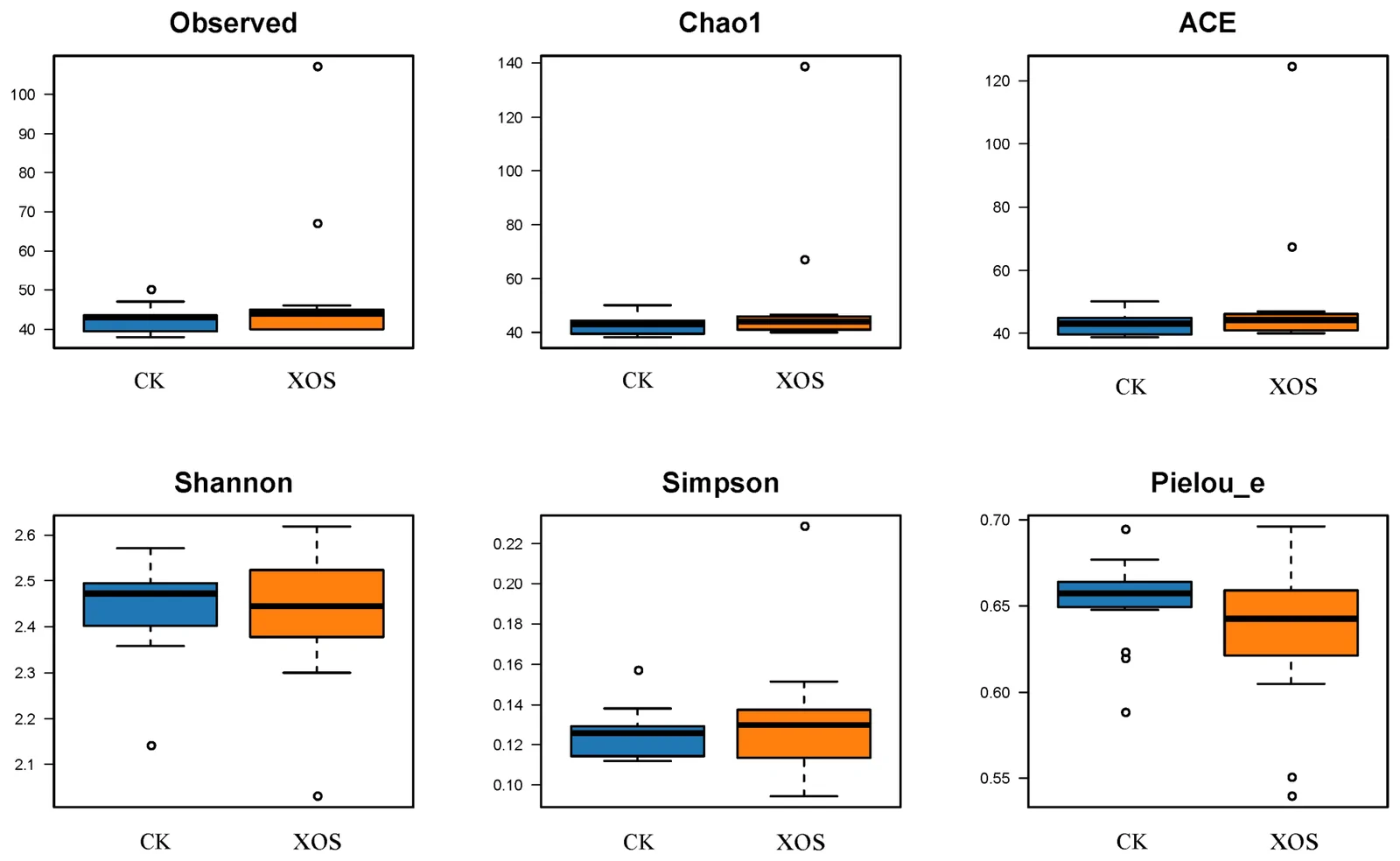
Effects of XOS supplementation on the alpha diversity of the honey bee gut microbiota.

To determine whether XOS supplementation altered the diversity of the honey bee gut microbiota, six alpha-diversity indices were compared between the CK and XOS groups. The richness-related indices, including Observed, Chao1, and ACE, showed similar distributions between the two groups, and no significant differences were detected (Figure 2, *p* > 0.05). Although several individuals in the XOS group showed relatively high richness values, the median values of these indices remained close to those of the control group.

The Shannon and Simpson indices, which reflect both taxon richness and abundance distribution, were also not significantly different between the CK and XOS groups (Figure 2, *p* > 0.05). Similarly, Pielou’s evenness did not differ significantly between treatments, despite a slightly lower median value and greater variation in the XOS group. Overall, these results showed that XOS supplementation did not produce a detectable change in bacterial richness, diversity, or evenness under the present experimental conditions.

### 3.3. XOS Supplementation Did Not Produce a Detectable Change in the Overall Gut Bacterial Community Structure

**Figure 3:**
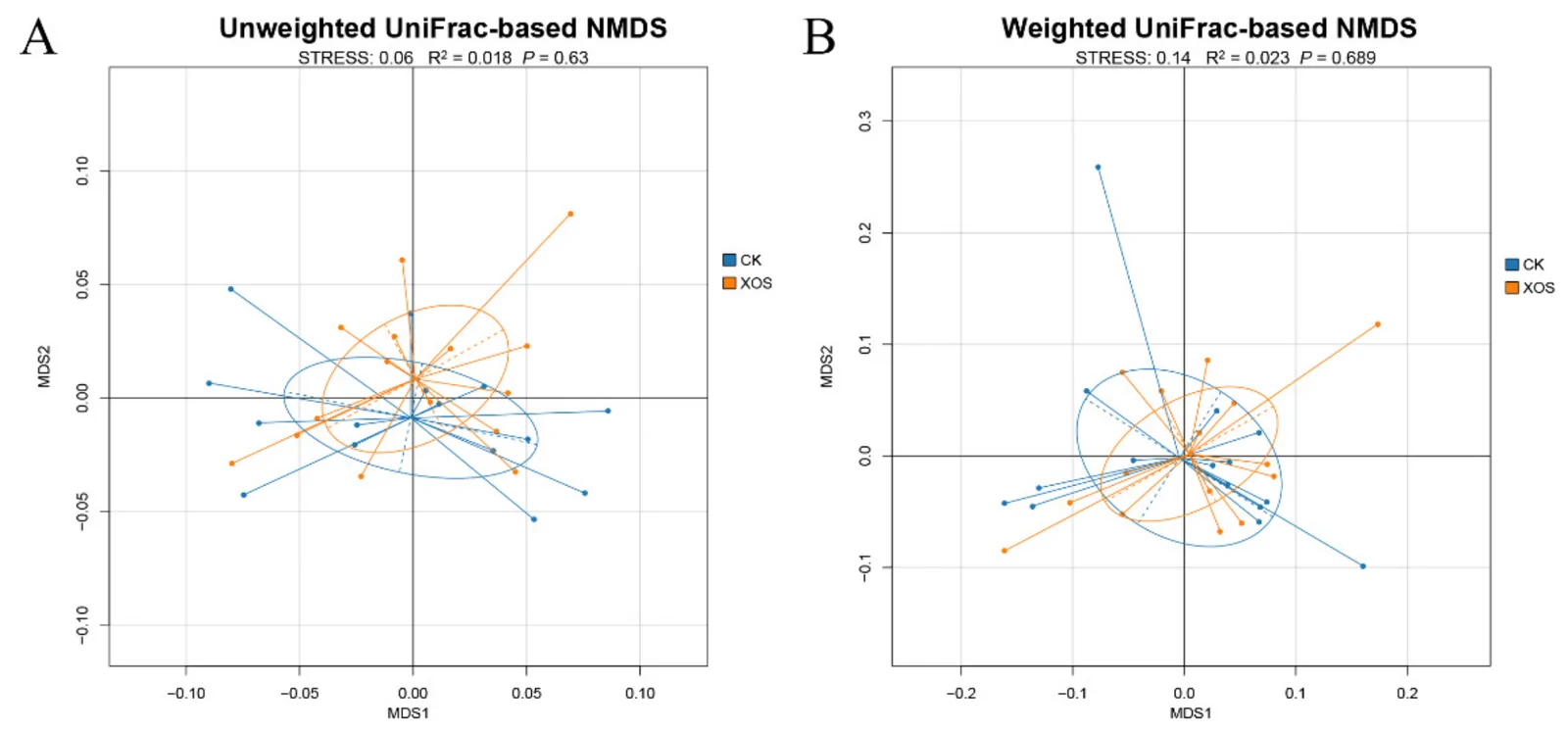
Effects of XOS supplementation on the beta diversity of the honey bee gut microbiota. NMDS analysis based on (**A**) unweighted UniFrac and (**B**) weighted UniFrac distances.

The effect of XOS supplementation on gut bacterial community structure was further examined using NMDS analysis based on unweighted and weighted UniFrac distances. In the unweighted UniFrac-based NMDS plot, samples from the CK and XOS groups were largely intermixed, with broad overlap between the two confidence ellipses (Figure 3A). PERMANOVA analysis showed that treatment explained only a small proportion of the variation in community composition and that the difference between groups was not significant (R^2^ = 0.018, *p* = 0.630). A similar pattern was observed in the weighted UniFrac-based NMDS analysis (Figure 3B). Samples from the two groups again showed substantial overlap, and no clear treatment-dependent clustering was observed. PERMANOVA confirmed that the relative abundance-weighted community structure did not differ significantly between the CK and XOS groups (R^2^ = 0.023, *p* = 0.689).

Together, these results indicate that XOS did not cause a detectable change in the overall abundance, diversity, or community structure of the honey bee gut microbiota.

### 3.4. XOS Supplementation Affected Several Predicted Microbial Functions

**Figure 4:**
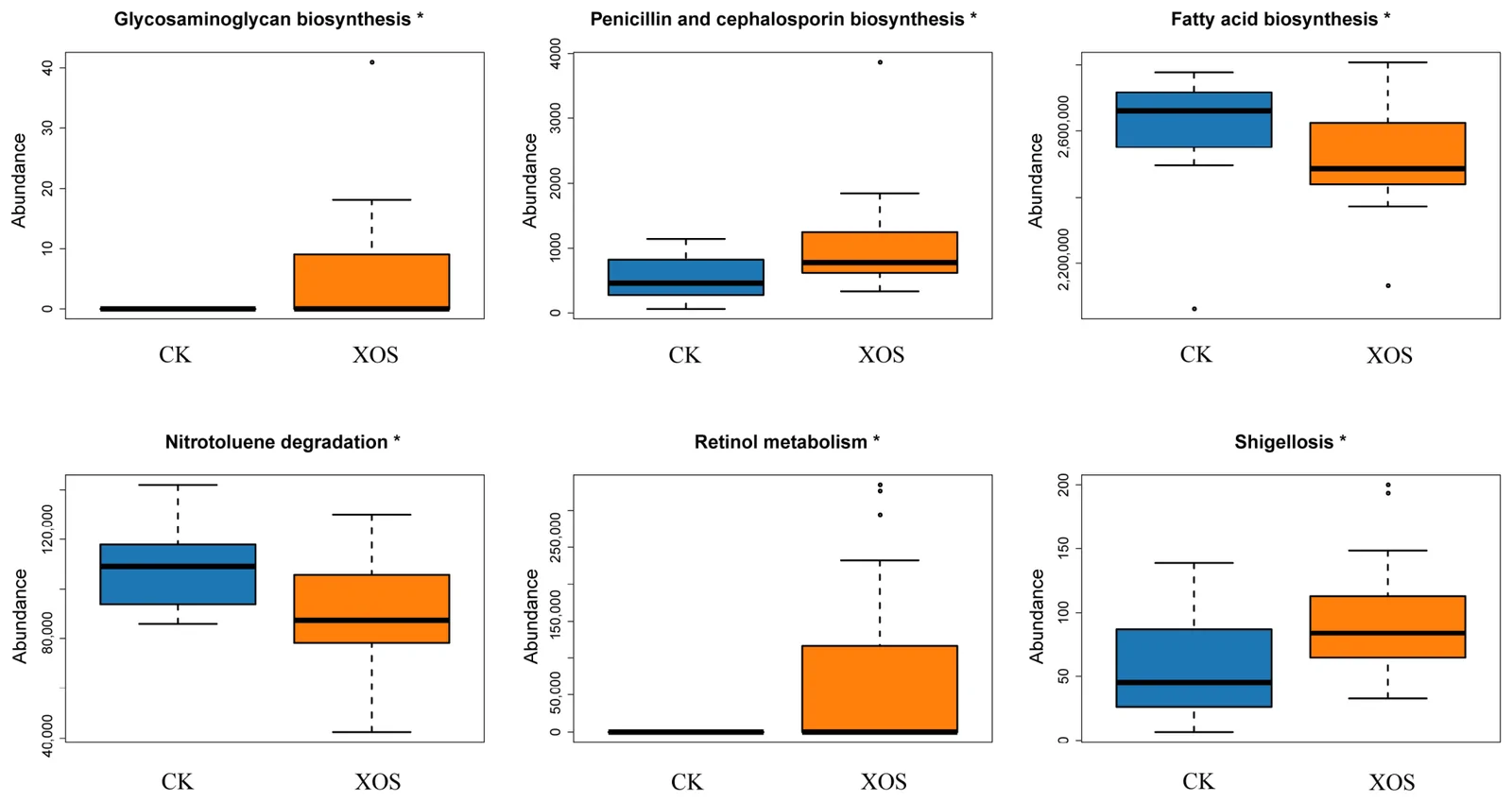
PICRUSt2-based prediction of significantly different microbial functional pathways between the CK and XOS groups. Asterisks indicate *p* < 0.05.

PICRUSt2-based functional prediction identified several pathways that differed between the CK and XOS groups. Compared with the CK group, the XOS group showed higher predicted abundances of pathways associated with glycosaminoglycan biosynthesis, penicillin and cephalosporin biosynthesis, retinol metabolism, and shigellosis, whereas fatty acid biosynthesis and nitrotoluene degradation showed lower predicted abundances (Figure 4, *p* < 0.05).

## 4. Discussion

XOS is commonly used as a prebiotic in humans to modulate intestinal microbiota [30]. However, in the present study, XOS did not cause a detectable change in the overall abundance, diversity, or community structure of the honey bee gut microbiota. These results suggest that the effects of XOS on the honey bee gut microbiota may be limited under the experimental conditions used here.

This result differs from the bifidogenic effects of XOS frequently reported in humans and livestock. XOS has been shown to selectively promote *Bifidobacterium* and alter microbial fermentation in both in vivo studies and in vitro bacterial culture experiments [32,38,39]. However, such effects cannot be directly extrapolated to honey bees, whose gut microbiota is composed of a small number of highly specialized bacterial lineages [16]. Previous work using an in vitro fermentation system derived from the gut microbiota of honey bee showed that different oligosaccharides produced distinct effects on bacterial growth and acid production [24]. By contrast, an earlier in vivo study of inulin supplementation in honey bee did not detect a clear prebiotic effect [25]. These observations suggest that the response to a given prebiotic may vary substantially among host species and experimental systems.

A possible explanation for the absence of a clear response is that the major honey bee gut bacteria may have a limited capacity to utilize XOS. The prebiotic effect of an oligosaccharide depends on the metabolic capacity of the resident microbiota, including the presence of suitable transport systems and carbohydrate-degrading enzymes [4,40,41]. Although XOS can be utilized by some mammalian-associated bifidobacteria, honey bee-associated *Bifidobacterium* and other core bacterial lineages may differ in their ability to metabolize this substrate [42,43]. The dietary environment may further influence this response, as honey bees consume readily available sugars and pollen-derived carbohydrates that may be preferentially utilized by gut bacteria. The availability of these alternative carbon sources, together with the spatial organization of the microbiota in the ileum and rectum [15,44], may reduce the selective advantage provided by XOS and limit its effects on individual bacterial groups. Nevertheless, because only one XOS concentration and one sampling time point were evaluated, possible dose- or time-dependent effects cannot be excluded. In addition, the relatively small number of replicate cages may have limited the statistical power to detect subtle treatment effects, and small biological effects may therefore have remained undetected.

Overall, this in vivo study showed that dietary XOS supplementation had only limited detectable effects on the abundance and structure of the honey bee gut microbiota. However, PICRUSt2 analysis indicated differences in several predicted microbial functional pathways, suggesting that XOS may influence the functional potential of the gut microbiota even in the absence of pronounced taxonomic changes. These differences should be interpreted cautiously because they were inferred from 16S rRNA gene data rather than directly measured microbial functions and require further validation using metagenomic or metabolomic approaches.

## 5. Conclusions

This in vivo study showed that dietary XOS supplementation had only limited detectable effects on the abundance, diversity, and community structure of the honey bee gut microbiota under the conditions tested. These findings indicate that XOS may have a limited capacity to modulate the gut bacterial community of *Apis mellifera* under the conditions tested. However, possible effects on microbial activity cannot be excluded, and further studies are needed to examine changes in microbial metabolism and host physiology. Given the increasing availability of commercial prebiotic products for honey bees, the efficacy of such products should be supported by host-specific in vivo evidence before widespread application.

## Data Availability

Raw sequencing data are publicly available in the Genome Sequence Archive at the China National Genomics Data Center under accession number CRA046674.
